# Thermal Mechanical Processing Effects on Microstructure Evolution and Mechanical Properties of the Sintered Ti-22Al-25Nb Alloy

**DOI:** 10.3390/ma9030189

**Published:** 2016-03-11

**Authors:** Yuanxin Wang, Zhen Lu, Kaifeng Zhang, Dalin Zhang

**Affiliations:** 1 School of Material Science and Engineering, Harbin Institute of Technology, Harbin 150001, China; luzhenhit@hit.edu.cn; 2 Department of Mechanical Engineering, Nanchang Vocation College, Nanchang 330004, China; trzhou@ncu.edu.cn

**Keywords:** Ti-22Al-25Nb alloy, thermal mechanical processing, mechanical properties, fracture mechanism

## Abstract

This work illustrates the effect of thermal mechanical processing parameters on the microstructure and mechanical properties of the Ti-22Al-25Nb alloy prepared by reactive sintering with element powders, consisting of O, B2 and Ti_3_Al phases. Tensile and plane strain fracture toughness tests were carried out at room temperature to understand the mechanical behavior of the alloys and its correlation with the microstructural features characterized by scanning and transmission electron microscopy. The results show that the increased tensile strength (from 340 to 500 MPa) and elongation (from 3.6% to 4.2%) is due to the presence of lamellar O/B2 colony and needle-like O phase in B2 matrix in the as-processed Ti-22Al-25Nb alloys, as compared to the coarse lath O adjacent to B2 in the sintered alloys. Changes in morphologies of O phase improve the fracture toughness (*K*_IC_) of the sintered alloys from 7 to 15 MPa·m^−1/2^. Additionally, the fracture mechanism shifts from cleavage fracture in the as-sintered alloys to quasi-cleavage fracture in the as-processed alloys.

## 1. Introduction

Owing to high specific strength and good oxidation resistance, Ti_2_AlNb alloys have received wide attention since it was discovered by Banerjee in the 1980s [1]. Great efforts have been made to develop the Ti_2_AlNb (based on O phase, orthorhombic phase) alloy with respect to preparation, microstructure and phases control, mechanical properties, and forming processes. Gogia and Nandy *et al.* [2,3] studied the relation of microstructure and mechanical properties of orthorhombic phase in the literature. They found that the morphology and scale of O phase had a significant effect on the tensile and creep properties. Wang *et al.* [4] investigated transformation of orthorhombic phase and B2 phase. They revealed phase transformation at different temperatures, and the effect of volume fraction of O phase on ductility. Further, Chen *et al.* [5,6] reported that heat treatment had an effect on the microstructure of the Ti-22Al-25Nb alloy. It was found that lamellar of O phase influenced yield strength and ductility. Generally, Ti_2_AlNb alloys had typical phases of O, B2/β (body centered cubic structure) and few α_2_-Ti_3_Al (ordered hexagonal closed packing structure). As a major phase, B2/β phase still played a key role in the properties of Ti_2_AlNb alloys. Boehlert [7,8] studied the effects of forging and rolling on microstructure in Ti-Al-Nb alloys. It was found that orthorhombic and B2/β phases had an effect on strength and ductility. The effects of alloying elements and heat treatment on fracture toughness of Ti-Al-Nb alloys were investigated by Kamat [9]. They found that higher Nb and lower Al were beneficial for improving the fracture toughness, and that fracture toughness increased with the volume fraction of B2/β phase. Many researchers also found that the strength and fracture toughness of the Ti_2_AlNb alloy depended strongly on phase compositions and morphologies [10,11,12]. Recently, the fabrication of the Ti-22Al-25Nb alloy via reactive sintering and hot pressing with element powders has been proposed due to its low cost. However, the sintered Ti-22Al-25Nb alloy had poor mechanical properties.

In this work, the as-sintered Ti-22Al-25Nb alloy was thermo-mechanically processed via isothermal forging. The influence of processing parameters on the microstructure evolution and mechanical properties of Ti-22Al-25Nb alloys were also investigated in order to better understand the mechanical behavior of the alloys and its correlation with the microstructural features.

## 2. Materials and Methods

**Figure 1:**
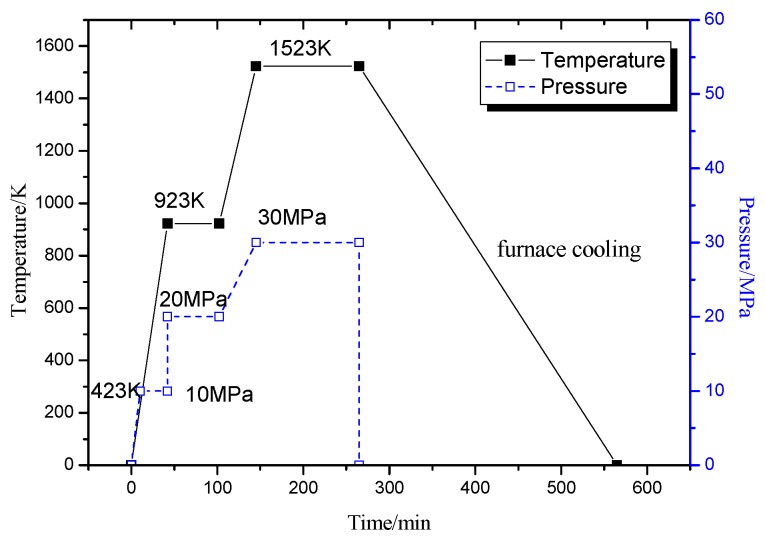
Time/temperature/pressure profile used in the reactive sintering of the mixed powders.

The Ti-22Al-25Nb alloy was prepared via reactive sintering and hot pressing with element powders. The as-mixed powders were sintered at 923 K for 20 MPa and 60 min, and then at 1523 K for 30 MPa and 120 min in a vacuum (10^−3^–10^−4^ torr, 1 torr = 1.33 × 10^2^ MPa) followed by furnace cooling. The profile of time/temperature/pressure used in the reactive sintering of the mixed powders is shown in Figure 1.

**Figure 2:**
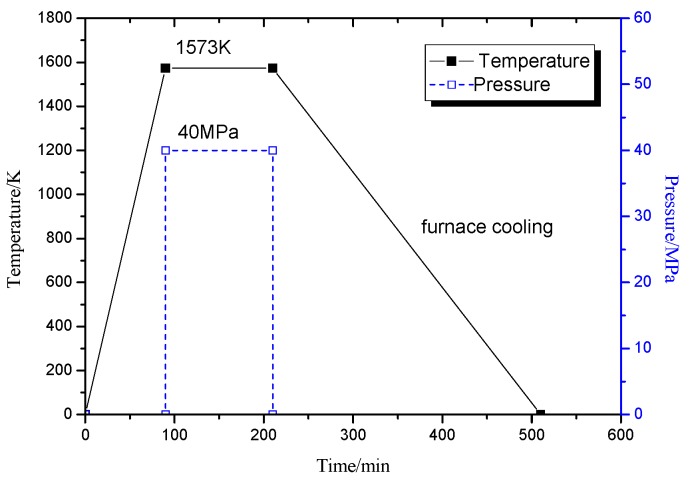
Time/temperature/pressure profile used in thermal mechanical processing.

The isothermal forging process was performed at 1573 K for 40 MPa and 60 min, followed by furnace cooling, as illustrated in Figure 2. The cylindrical compression specimens, with a size of 8 mm × 12 mm, were cut from the sintered alloy. The axial of cylindrical compression specimens was along the hot pressing direction. Both of the end surfaces of specimens were grinded and polished. Isothermal compression tests were conducted in a Gleeble-1500D thermal simulator (DSI, St. Paul, MN, USA) using a strain rate range within 0.001–0.005 s^−1^ to a height reduction of 70%. The graphite molds were used during the isothermal forging process.

Specimens for microstructural examinations were prepared using standard metallographic techniques. A Quanta 200FEG scanning electron microscope (FEI Co., Inc., Hillsboro, OR, USA) was used for the examination of the microstructures and the fracture surfaces of the failed specimens. A Tecnai G2F20 transmission electron microscope (TEM, FEI Co., Inc.) was used for investigations of the structure of the phases. The TEM foils were prepared by electrochemical dissolution in a twin-jet electropolishing unit using a solution consisting of 60% methanol, 34% buthanol and 6% perchloric acid.

The tensile tests were conducted at room temperature on an Instron-5500 machine at a constant crosshead speed of 0.05 mm/min (approximate strain rate is 1.0 × 10^−4^ s^−1^). Tensile specimens have gauge dimensions of 8 mm × 3 mm × 2 mm, and a total of five specimens were tested for each material. It should be noted that the strain gauges were used in the tensile testing in order to measure accurately. The ultimate tensile strength (UTS) and total elongation (EL) were determined from the stress-strain curve derived from the force-displacement data.

Estimations of fracture toughness of the specimens were carried out using the single-edge notch beam (SENB) method on an Instron-6900 machine at a speed of 0.05 mm/min. The samples for the fracture toughness test were machined with a size of 2 mm × 4 mm × 16 mm. The fracture surfaces were analyzed using a scanning electron microscope (SEM).

## 3. Results and Discussion

### 3.1. Tensile Behaviors

**Figure 3:**
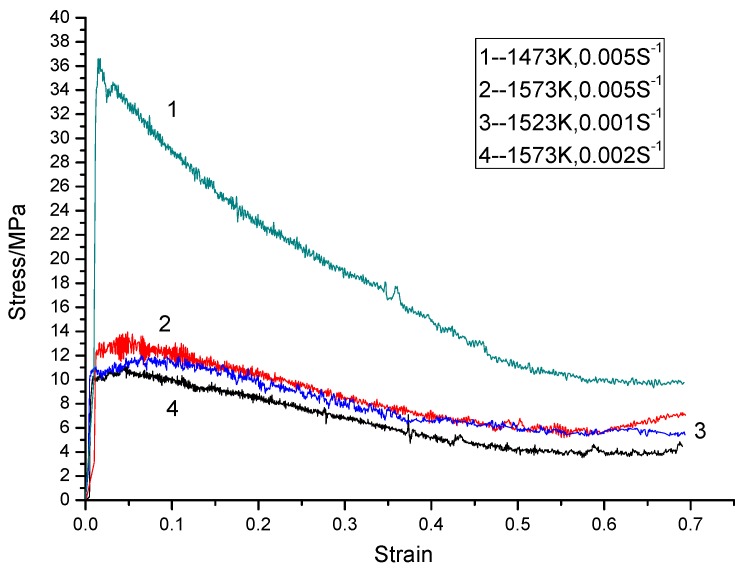
True stress-strain curves for the sintered Ti-22Al-25Nb alloy at different deformation temperatures and different strain rates.

Figure 3 shows the true stress-strain curves of the sintered Ti-22Al-25Nb alloy at different deformation temperatures and strain rates. As can be seen from Figure 3, flow stress increases rapidly to a peak value, and is then followed by a gradual decreasing until a steady flow in each curve is displayed. The profile of the stress-strain curve is likely due to a dynamic balance between work hardening and flow softening. Flow softening could be caused by dynamic recrystallization (DRX) [13]. Additionally, Figure 3 depicts that flow stress decreases with temperature increasing and strain rate decreasing. The peak of flow stress reduces from 36 to 14 MPa (or 11 MPa), as deformation temperature increases from 1473 to 1523 K (or 1573 K). Interestingly, flow stress decreases remarkably at a strain rate of 0.005 s^−1^ from 1473 to 1573 K, as illustrated by the comparison between curves 1 and 2 in Figure 2, indicating that the sensitivity of flow stress was related to deformation temperature.

**Figure 4:**
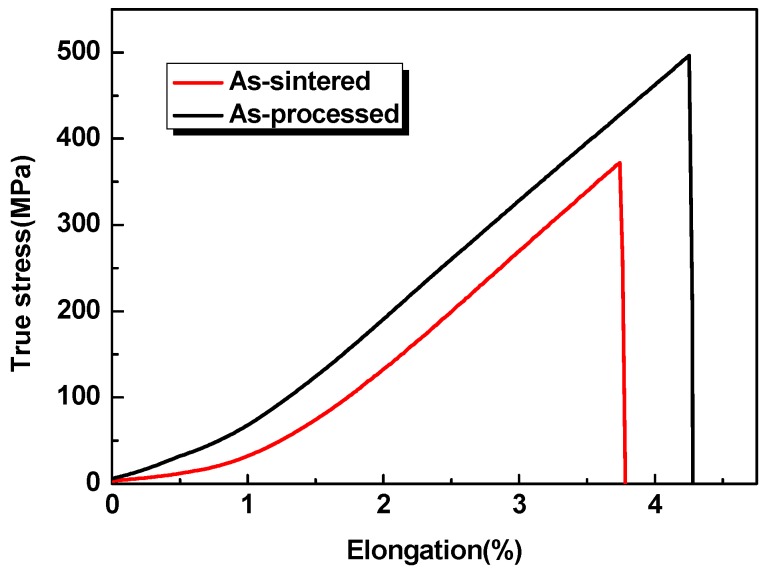
Room temperature tensile curves of the Ti-22Al-25Nb alloy.

Figure 4 illustrates room temperature tensile curves of the Ti-22Al-25Nb alloy, as-sintered and as-processed. As shown in Figure 4, both the as-sintered and as-processed alloys experience no yield behavior before fracture. Tensile strength of the as-sintered alloy was only 340 MPa, and elongation was about 3.6%. However, for the sintered Ti-22Al-25Nb alloy processed by isothermal forging, tensile strength increases up to 500 MPa, and elongation is approximately 4.2%.

### 3.2. Microstructure Characterization

**Figure 5:**
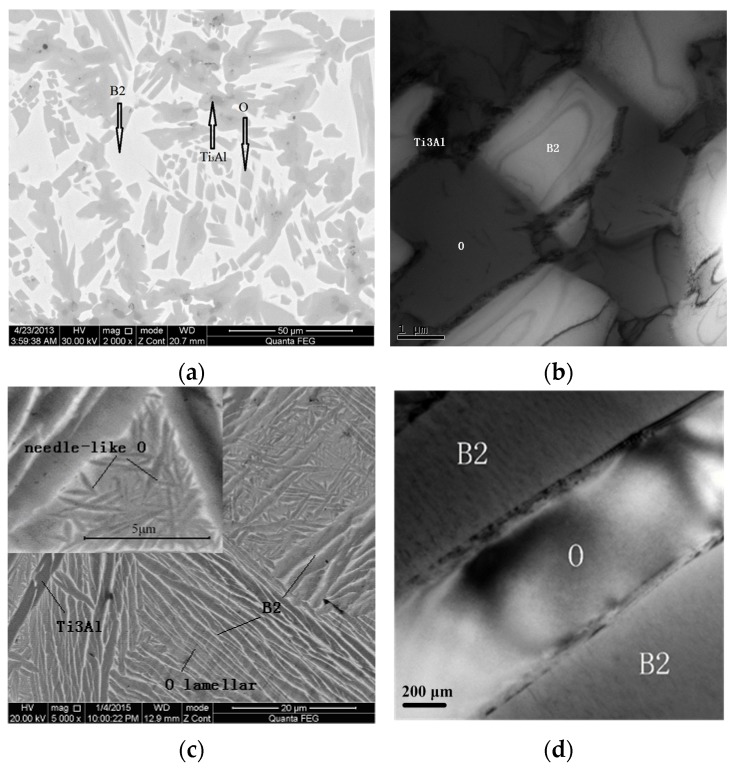
Microstructure of the Ti-22Al-25Nb alloy: (**a**) SEM and (**b**) TEM image of as-sintered alloy; (**c**) SEM and (**d**) TEM image of as-processed alloy.

Figure 5 shows the microstructure of the Ti-22Al-25Nb alloy, as-sintered and as-processed. As shown in Figure 5a, the microstructure contains the B2 matrix, equiaxial Ti_3_Al or O phase, and the coarse lath O/rim O phase. The distribution of O and Ti_3_Al is non-homogeneous. The rim-O around the Ti_3_Al particle, was formed by the peritectoid reaction of B2 and Ti_3_Al phases [14]. Therefore, the size of coarse primary Ti_3_Al can be reduced with the peritectoid reaction, even fading away to transform into O phase completely. However, as can be seen from Figure 5b, the microstructure consists mainly of lamellar O and B2, needle-like O and lath Ti_3_Al after isothermal forging of the as-processed alloys. The lamellar O and B2 phase occurs alternately. Dislocations in the interface of the two phases were detected under TEM observation, as shown in Figure 5b,d. Due to the difference of crystal lattice in the two, dislocation is a possible way for a transition between the O and B2 phases. As to the developed mechanism of O phase [15,16], the discontinuous precipitating of O lamellar results in B2/O dispersing alternately.

Isothermal forging apparently improves homogeneity of the sintered Ti-22Al-25Nb alloy. O and Ti_3_Al phases transform into B2 phase when the processing temperature is above the B2 transus temperature. Under a slow cooling, O and Ti_3_Al reprecipitate from the B2 matrix. Under the condition of thermal mechanical processing, the load promotes the resolving of O phase, accelerating phase transformation. In addition, the compactness of the Ti-22Al-25Nb sintered alloy is also improved, and the defects, such as holes, are reduced as much as possible. For example, at the initial sintering course, the pores being caused by the diffusion factor (caused by the Kirkendall effect), is closed in the matrix, and the pores are then seamed under compressing stress.

As depicted in Figure 5c, O phases disperse homogeneously within the B2 matrix, which is suggested to be one reason for the better tensile properties of the as-processed alloy, as compared to the as-sintered alloy. Additionally, as mentioned above, the volume fraction of O phase increases significantly in the as-processed alloy. Therefore, the improved strength is also likely due not only to the disperse distribution of the fine needle-like O precipitation in the B2 matrix, but also to the O/B2 lamellar colony. Our results agree well with results in the literature. It has been widely proved that the tensile behavior of the Ti_2_AlNb-based alloys is highly dependent on microstructural characteristics, especially on phase compositions and the volume fraction of each phase [17]. Wang *et al.* [14] reported that the strength of Ti-22Al-25Nb alloys could vary with the volume fraction of each phase, morphologies, and distribution structure. Boehlert [18] studied grain size effect on the strength and ductility. It was proved that coarse B2 phase still exhibited mixed intergranular and transgranular fracture and cleaved grains. Peng *et al.* believes that, with B2 grain size obeying the Hall-Petch relation, the coarse prior B2 phase would deteriorate the strength and ductility at room temperature [19,20]. Therefore, the coarse B2 grain in the as-sintered alloy displayed a brittle fracture, with a strength of only 340 MPa.

### 3.3. Fracture Toughness and Fracture Mechanism Analyses

The results of fracture toughness tests reveal that the *K*_IC_ value for the as-sintered alloy is only 7 MPa·m^−1/2^, while the *K*_IC_ value for the as-processed alloy increases up to 15 MPa·m^−1/2^. The variation of fracture toughness values between 7 and 15 MPa·m^−1/2^ was prone to depend on the microstructure. The interfaces in the transformed B2 phase, not the grain size, predominantly control fracture toughness in orthorhombic alloys [21].

**Figure 6:**
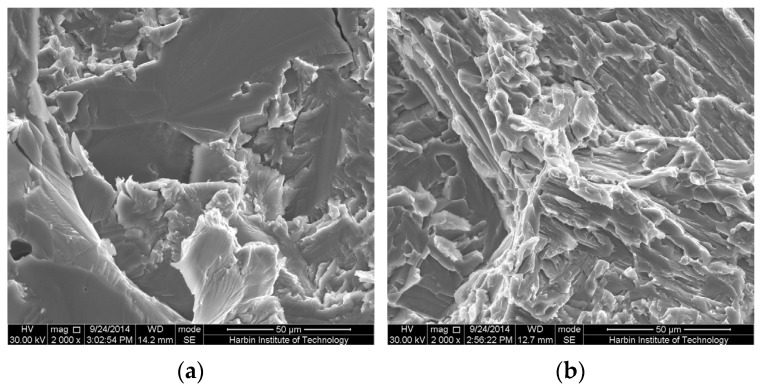
SEM images of the fracture surfaces for samples after tensile: (**a**) as-sintered alloy and (**b**) as-processed alloy.

Figure 6 shows SEM images of the fracture surfaces for samples after tensile. As shown in Figure 6a, the fractography of river pattern, secondary cracks and pores can be observed and indicates a typical cleavage fracture. However, tearing ridges were formed in the as-processed sample, as depicted in Figure 6b, indicating a quasi-cleavage fracture. It is suggested that a slight plastic deformation has been developed in the as-processed alloy. A slight plastic deforming deriving from ductile tearing between different microcracks propagation decreases the stress concentration of the cracking tip to blunt cracking propagation. If the cracking propagation continues to develop, it is necessary to enhance the stress. Hence, this results in an increase in strength.

Apparently, the deformation causes dislocations to glide. Dislocation gliding in a completed order crystal is as easy as that in the disordered alloy. However, a low degree of order and a high defect density could result in degrading the dislocations’ movability owing to the variation of the dislocation moving path, further deteriorating the plasticity and ductile. According to Stroh’s cracking nucleation theory [22], although a nominal stress is still exhibited at a lower level, a higher local stress concentration can be developed. The pile-up dislocations around obstacles could result in local stress concentration, and the cracks could then be initiated, while a higher local stress concentration would not be released by means of plastic deforming.

The structure of the lower symmetry O phase is closely related to the Ti_3_Al ordered hexagonal structure and the very small lattice parameter differences between O and Ti_3_Al. Therefore, the slip systems in O phase are analogous to that of Ti_3_Al [23]. In fact, dislocations gliding could be dependent on not only Burgers vectors, but the energies of faults, such as an APB (antiphase boundary) or a SISF (superlattice intrinsic stacking fault). The Cottrell atmosphere accounts for the interaction of dislocations that belong to two intersecting slip planes. Usually, Cottrell’s theory holds that dislocation reactions could be prone to make the sessile dislocation form in a body-centered cubic (BCC)-type alloy. Further, the sessile dislocations accumulate up to a critical value to transform into a microcrack. Dislocations were dissociated into two superpartials in their slip planes and were separated by an antiphase boundary (APB) or faults [24,25]. A consistent dislocations slip would be determined by a dislocation reaction depending on an antiphase boundary or faults. The energies of the resultant dislocations are lower than those before the reactions. For example, the superpartial dislocations are prone to developing the resultant dislocations in order to decrease the energies. While the reactions to make the sessible dislocations were proposed, a microcrack could be developed at the sessible dislocations so that the plastic deformation was deteriorated. Bannered [26] studied dislocation reactions of O or Ti_3_Al and observed them at room temperature, not seen at high temperature. It was also proved that the superdislocations were pinned through a dislocation reaction. In conclusion, an intrinsic characteristic of dislocation slip in Ti_2_AlNb alloys, derived from large slip vectors in ordered lattice and dissociations of superdislocations, would result in its poor plasticity at room temperature.

Generally, it is believed that fracture toughness depends on the microstructure and morphology, e.g., the size of secondary O-lamellar within B2 phase. The coarse B2 and O in the as-sintered Ti-22Al-25Nb alloy facilitate not only cracking nucleation but also cracking propagation. As mentioned above, fracture toughness was controlled by the interfaces of different phases instead of the grain size. A tortuous cracking propagation path was controlled by the interfaces between O and B2 and Ti_3_Al. Therefore, the lamellar O/B2 colony could decrease the rate of cracking propagation, and further enhance the fracture toughness [27].

## 4. Conclusions

In this paper, the Ti-22Al-25Nb alloy was prepared by reactive sintering and hot pressing with element powders. The sintered Ti-22Al-25Nb alloy was treated using isothermal forging in order to improve the microstructure. The mechanical properties were investigated at room temperature, and the mechanism was analyzed. The following conclusions were drawn in this investigation:

1. The sintered Ti-22Al-25Nb alloy was processed by isothermal forging. The microstructure was homogenous compared to the as-sintered alloy. The morphology of O phase changed from the coarse lath O to the lamellar O and acicular O. The distribution of O phase was relatively homogeneous.
2. While the sintered alloy was processed by isothermal forging, the tensile strength and elongation increased from 340 MPa, 3.6% to 500 MPa, 4.2%, respectively. In addition, the fracture toughness (*K*_IC_) was enhanced from 7 to 15 MPa·m^−1/2^.
3. Judging by the fractography observations, a typical embrittlement fracture consisting of cleavage and quasi-cleavage fracture was developed. The morphologies of O phase within B2 phase had a key effect on improving strength and ductile. The fracture mechanism changing from cleavage fracture to quasi-cleavage fracture mainly resulted from morphologies and the distribution of O phase. Meanwhile, the interfaces in the transformed O/B2 colony, not the grain size, predominantly controlled fracture toughness in the Ti-22Al-25Nb alloy.
